# Surgical Outcomes of Open and Laparoscopic Hartmann Reversal: A Single-Center Comparative Study

**DOI:** 10.7759/cureus.67102

**Published:** 2024-08-18

**Authors:** Mu-Han Tsai, Ming-Jenn Chen, Khaa-Hoo Ong, Chih-Ying Lu, Chung-Han Ho, Hsuan-Yi Huang, Yu-Feng Tian, I-Ning Yang

**Affiliations:** 1 Department of General Surgery, Chi Mei Medical Center, Tainan, TWN; 2 Department of Research, Chi Mei Medical Center, Tainan, TWN; 3 Department of Colorectal Surgery, Chi Mei Medical Center, Tainan, TWN; 4 Department of Nephrology, Chi Mei Medical Center, Tainan, TWN

**Keywords:** laparoscopic hartmann reversal, open hartmann reversal, colostomy, laparoscope, hartmann reversal, hartmann’s operation

## Abstract

Background and objectives: Hartmann reversal (HR) is challenging and traditionally requires a large laparotomy wound. With the development of minimally invasive techniques, laparoscopic reversal of Hartmann's operation (HO) was attempted. We aimed to evaluate the outcomes of laparoscopic Hartmann reversal (LHR) versus open Hartmann reversal (OHR).

Materials and methods: In this study, we included 33 patients who underwent HR at Chi Mei Medical Center between January 2015 and March 2023. Ten patients received LHR, while 23 received OHR. We compared patient demographics, perioperative outcomes, early postoperative complications, and late postoperative complications between the two groups.

Results: There was no significant difference in the baseline demographics of both groups. Compared to the open method, the LHR group had a shorter hospital stay and time to solid diet. The median length of hospital stay in the OHR and LHR groups was 15.00 (Q1-Q3: 13.00-16.00) and 11.5 (Q1-Q3: 10.00-14.00) days (p = 0.028), respectively. The median time to solid diet was 8.00 (Q1-Q3: 7.00-8.00) days in the OHR group and 5.00 (Q1-Q3: 5.00-7.00) days in the LHR group (p = 0.022). No statistical significance between the groups was noticed in early and late postoperative complications.

Conclusions: Whether using a laparoscopic or an open method, HR is challenging. In our study, patients who underwent LHR were associated with reduced hospital stays and faster bowel movements.

## Introduction

Hartmann's operation (HO) is a surgical procedure that involves removing the rectosigmoid colon. This procedure includes closing the lower end of the rectum (anorectal stump) and creating a stoma on the abdominal wall [1,2]. It is typically used in emergencies where there is a left-sided colon perforation or obstruction, caused by diverticulitis, ischemic colitis, intestinal tumors, or malignancies [2,3]. The advantages of this surgery are that it reduces surgical time and lowers the risk of leakage caused by intestinal anastomosis. However, a significant drawback is a decrease in the patient's quality of life due to having an intestinal stoma [1,4].

When the patient's condition stabilizes postoperatively, the surgeon will evaluate the closure of the stoma, also known as Hartmann reversal (HR). In the past, HR was usually performed through open abdominal surgery. However, with the advancement of laparoscopic techniques, some surgeons have considered using laparoscopy to close such stomas. According to current research, laparoscopic Hartmann reversal (LHR) yields lower postoperative pain scores, shorter hospital stays, and quicker bowel recovery than open Hartmann reversal (OHR) [1]. Nevertheless, some studies indicate no significant difference in short-term surgical outcomes between laparoscopic and open surgery [5].

As minimally invasive surgery gains emphasis on smaller incisions, some surgeons at our institution have adopted laparoscopic methods for stoma closure. However, no research has compared the patient selection and postoperative and long-term complications between laparoscopic and traditional open abdominal surgeries in our institution. Hence, we retrospectively analyzed patients who underwent HO at Chi Mei Medical Center, comparing the outcomes of stoma closure surgeries using laparoscopy and traditional open surgery. This study aims to provide surgeons with valuable information when explaining to patients the advantages, disadvantages, and differences between the two surgical approaches.

## Materials and methods

Data collection methods

**Figure 1:**
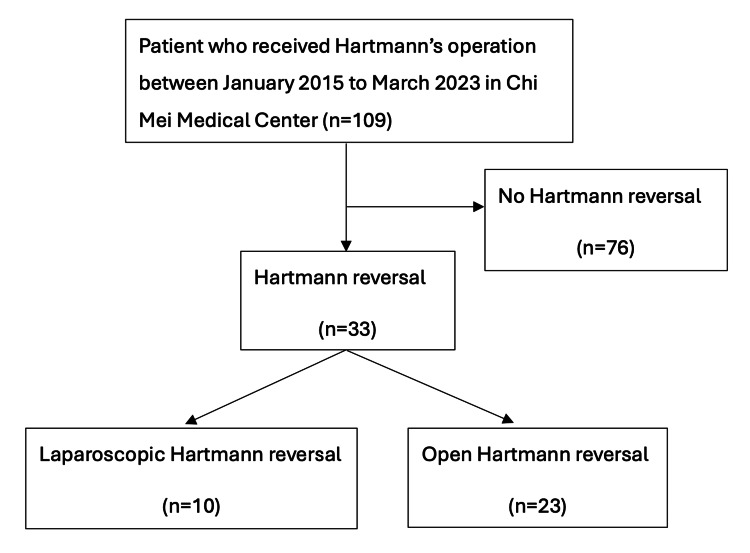
Flow chart of patient selection.

This is a retrospective, single-center study. Between January 2015 and March 2023, 109 patients received elective or emergent HO at Chi Mei Medical Center. Among these 109 patients, 33 underwent HR. Ten patients received LHR, while 23 received OHR, as shown in Figure 1. This study was approved by the Institutional Review Board of Chi Mei Medical Center (IRB No.: 11303-002) and conducted according to the principles of the Declaration of Helsinki.

Surgical procedures

Preoperatively, patients underwent comprehensive examinations, such as a lower gastrointestinal series with contrast medium, abdominal computed tomography scan, or colonoscopy. If there was no leakage, stump dehiscence, or other contraindications, an operation with stoma closure was arranged. Before the operation, colon preparation was necessary for all of the patients. The medical team would educate the patient two days before the surgery to take a low-fiber diet. Bowel cleaning agents were given on the day before the operation. All patients who received surgery were placed in a lithotomy position under general anesthesia. First, an incision was made at the mucocutaneous border of the end stoma. We gently mobilized the end stoma by sharp dissection until it entered the peritoneal cavity. The stoma was then taken down into the abdominal cavity. We will discuss the subsequent steps of the two groups separately.

Laparoscopic Hartmann Reversal

After putting the end stoma into the abdominal cavity, the anvil head of a circular stapler was introduced into the end side of the stoma, and a purse string suture of 2-0 Prolene was made. A 12 mm trocar was inserted via the previous stoma wound. The wound was closed with 1-0 Vicryl to spare space for one trocar. The pneumoperitoneum was then established. One 12 mm trocar was inserted through the umbilicus under the scope guide. Another 12 mm trocar and 5 mm trocar were inserted through the right lower and right upper abdomen, respectively. The surgeon and the scopist stood on the patient's right side, with the surgeon toward the patient's foot and the scopist toward the patient's head. Furthermore, the assistant stood on the patient's left side.

First, adhesiolysis was performed. Then, the rectal stump was identified and isolated from the pelvic cavity. If the rectal stump and proximal descending colon (or sigmoid colon) were redundant, side-to-side or end-to-side anastomosis would be performed. If the bowels were close to each other, end-to-end anastomosis was first considered. An endoscopic linear stapler was applied for side-to-side anastomosis, and a circular stapler was utilized for end-to-side anastomosis. A Jackson-Pratt (J-P) drain was put in the pelvis. However, surgeons may make adjustments based on clinical conditions.

Open Hartmann Reversal

After putting the end stoma into the peritoneum cavity, it was resected segmentally as proximal to the end as possible. A laparotomy wound was made through the abdominal midline. Adhesiolysis was conducted to free the distal stump of the sigmoid colon or rectum, which was located in the pelvic outlet. Then, anastomosis was performed (end-to-side, end-to-end, or side-to-side, depending on different conditions). Some surgeons prefer covering the suture line with the omentum. A J-P drain was put at the pelvis. Some surgeons put Penrose in the subcutaneous space of the previous stoma wound.

Surgical outcomes

We analyzed surgical outcomes and classified them into perioperative outcomes, early postoperative complications, and late postoperative outcomes. Patients’ data, including age, gender, body mass index (BMI), American Society of Anesthesiologists (ASA) classification, comorbidities, initial indications for HO, and median interval before HR, were analyzed. Perioperative outcomes such as operation time, blood loss, postoperative pain score, length of hospital stay, and time to solid diet were evaluated. We assessed pain scores using the Visual Analog Scale (VAS) on postoperative days zero, one, three, and seven. The highest score was collected on postoperative days zero, one, three, and seven, and we compared the average. Early postoperative complications were defined as events within six months after the operation, while late postoperative complications were defined as events occurring over six months.

Statistical analysis

Descriptive statistics were used to present the means and standard deviations (SD) or medians and interquartile ranges for continuous variables, and numbers and percentages for categorical variables. There were no missing data. The perioperative surgical outcomes, such as operation time, blood loss, and pain score, were measured on a continuous scale. Early and late postoperative complications were measured on a categorical scale. We used the Student’s t-test to compare continuous variables and Fisher’s test for categorical variables. The Wilcoxon rank sum test was applied to compare median (Q1-Q3) variables, including length of hospital stay and time to solid diet. A p-value of less than 0.05 was considered statistically significant in this study. Statistical analyses were performed using SPSS version 25 (IBM Corp., Armonk, NY).

## Results

Baseline characteristics

From January 2015 to March 2023, 33 patients received HR at Chi Mei Medical Center. A total of 23 patients underwent OHR, while 10 patients underwent LHR. The demographic characteristics of the open and laparoscopic groups are shown in Table 1. The two groups showed no statistical difference in terms of age, gender, BMI, ASA, and comorbidities. The mean age was 68.65 ± 12.04 years in the OHR group and 61.40 ± 14.40 years in the LHR group (p = 0.1439). The mean BMI was 23.87 ± 3.74 kg/m^2^ and 23.08 ± 2.27 kg/m^2^ in the OHR and LHR groups (p = 0.5399), respectively.

**Table 1:** Baseline demographic data of all the patients. Student’s t-test was used for continuous variables; Fisher’s test was used for categorical variables; Wilcoxon rank sum test was used for median variables. Data were considered statistically significant when p < 0.05. BMI: body mass index; ASA: American Society of Anesthesiologists; HR: Hartmann reversal; OHR: open Hartmann reversal; LHR: laparoscopic Hartmann reversal.

	All patients (n = 33)	OHR group (n = 23)	LHR group (n = 10)	p-value
Age (years) (mean ± SD)	66.45 ± 13.02	68.65 ± 12.04	61.40 ± 14.40	0.1439
Gender, n (%)				1.0000
Male	16 (48.48)	11 (47.83)	5 (50.00)	
Female	17 (51.52)	12 (52.17)	5 (50.00)	
BMI (kg/m^2^) (mean ± SD)	23.63 ± 3.35	23.87 ± 3.74	23.08 ± 2.27	0.5399
ASA classification, n (%)				0.5672
II	4 (12.12)	2 (8.70)	2 (20.00)	
III	29 (87.88)	21 (91.30)	8 (80.00)	
Comorbidities, n (%)				
Cardiovascular disease	25 (75.76)	18 (78.26)	7 (70.00)	0.6728
Diabetes mellitus	7 (21.21)	3 (13.04)	4 (40.00)	0.1605
End-stage renal disease	2 (6.06)	2 (8.70)	0 (0.00)	1.0000
Autoimmune disease	3 (9.09)	1 (4.35)	2 (20.00)	0.2117
Liver cirrhosis	2 (6.06)	2 (8.70)	0 (0.00)	1.0000
Human immunodeficiency virus	1 (3.03)	0 (0.00)	1 (10.00)	0.3030
Cause of initial operation, n (%)				1.0000
Benign	30 (90.9)	21 (91.32)	9 (90.0)	
Malignant	3 (9.1)	2 (8.7)	1 (10)	
Median interval duration before HR (months) (mean ± SD)	10.23 ± 16.97	12.46 ± 19.97	5.10 ± 2.60	0.259

The comorbidities included cardiovascular disease, diabetes mellitus, end-stage renal disease, autoimmune disease, liver cirrhosis, and human immunodeficiency. The prevalence of these comorbidities in both groups was comparable. The median interval duration before HR was 10 months, with no statistical significance between the two groups.

Perioperative outcomes

For the perioperative outcomes, the two groups exhibited statistical significance in length of hospital stay and time to solid diet, as shown in Table 2. The median length of hospital stay in the OHR and LHR groups was 15.00 (Q1-Q3: 13.00-16.00) and 11.5 (Q1-Q3: 10.00-14.00) days (p = 0.028), respectively. The median time to solid diet was 8.00 (Q1-Q3: 7.00-8.00) days in the OHR group and 5.00 (Q1-Q3: 5.00-7.00) days in the LHR group (p = 0.022). There were no significant differences when comparing operation time, blood loss, and postoperative pain score.

**Table 2:** Perioperative outcomes. Student’s t-test was used for continuous variables; the Wilcoxon rank sum test was used for median (Q1-Q3) variables. Data were considered statistically significant when (*) p < 0.05. OHR: open Hartmann reversal; LHR: laparoscopic Hartmann reversal.

	All patients (n = 33)	OHR group (n = 23)	LHR group (n = 10)	p-value
Operation time (minutes)	255.15 ± 85.03	253.04 ± 76.33	260.00 ± 106.9	0.833
Blood loss (ml)	133.33 ± 145.06	152.17 ± 150.36	90.00 ± 128.67	0.2644
Postoperative pain score	3.92 ± 1.95	4.05 ± 2.05	3.60 ± 1.77	0.5487
Length of hospital stay (day)	14.00 (12.00-16.00)	15.00 (13.00-16.00)	11.50 (10.00-14.00)	0.028*
Time to solid diet (day)	7.00 (6.00-8.00)	8.00 (7.00-8.00)	5.00 (5.00-7.00)	0.022*

Early postoperative complications

For early postoperative outcomes, as shown in Table 3, we evaluated the patients who suffered from wound infection, intra-abdominal infection, ileus, upper gastrointestinal bleeding, and lower gastrointestinal bleeding in the OHR and LHR groups. Nine patients (39.13%) in the OHR group and two patients (20%) in the LHR group suffered from any of the above-mentioned complications, indicating no significant difference (p = 0.682).

**Table 3:** Early postoperative complications. Fisher’s test was used for categorical variables. Data were considered statistically significant when p < 0.05. OHR: open Hartmann reversal; LHR: laparoscopic Hartmann reversal.

	All patients (n = 33)	OHR group (n = 23)	LHR group (n = 10)	p-value
Early postoperative complications	11 (33.33)	9 (39.13)	2 (20.00)	0.682
Type of early postoperative complications, n (%)				1.000
Wound infection	2 (6.06)	2 (8.70)	0 (0.00)	
Intra-abdominal infection	0 (0.00)	0 (0.00)	0 (0.00)	
Ileus	3 (9.10)	2 (8.70)	1 (10.00)	
Lower gastrointestinal bleeding	2 (6.06)	2 (8.70)	0 (0.00)	
Upper gastrointestinal bleeding	3 (9.10)	2 (8.70)	1 (10.00)	
Others	2 (6.06)	2 (8.70)	0 (0.00)	

Late postoperative complications

Table 4 shows late postoperative complications. Seven patients (30.4%) in the OHR group and four patients (40%) in the LHR group were recorded to have late postoperative complications (p = 0.696). The most frequently encountered postoperative complication was an incisional hernia, which was composed of five patients (21.74%) in the OHR group and three patients (30%) in the LHR group.

**Table 4:** Late postoperative complications. Fisher’s test was used for categorical variables. Data were considered statistically significant when p < 0.05. OHR: open Hartmann reversal; LHR: laparoscopic Hartmann reversal.

	All patients (n = 33)	OHR group (n = 23)	LHR group (n = 10)	p-value
Late postoperative complications	11 (33.33)	7 (30.43)	4 (40.00)	0.696
Type of late postoperative complications, n (%)				
Incisional hernia	8 (24.24)	5 (21.74)	3 (30.00)	0.673
Ileus	1 (3.03)	1 (4.35)	0 (0.00)	1.000
Anastomosis stricture	1 (3.03)	0 (0.00)	1 (10.00)	0.303
Ulcerative colitis flare-up	1 (3.03)	1 (4.35)	0 (0.00)	1.000

Clinical information of patients with incisional hernias

Table 5 provides clinical information on eight patients with incisional hernias, including the location of the hernias and the methods used for hernia repair. Cases 1-5 were from the OHR group, and cases 6-8 were from the LHR group. The average age of cases 1-5 was 70.20 ± 17.72 years and 63.33 ± 6.94 years for cases 6-8. Patients with incisional hernias were those of older age in the OHR (70.20 ± 17.72 vs. 68.65 ± 12.04 years) and LHR (63.33 ± 6.94 vs. 61.40 ± 14.40 years) groups. The average BMI in cases 1-5 and 6-8 was 27.92 ± 2.97 kg/m^2^ and 23.99 ± 1.85 kg/m^2^, respectively. Patients undergoing OHR with incisional hernias had a higher mean BMI (27.92 ± 2.97 vs. 23.87 ± 3.74 kg/m^2^), while those receiving LHR had a comparable BMI (23.99 ± 1.85 vs. 23.08±2.27 kg/m^2^).

**Table 5:** Clinical information of patients with incisional hernias. Cases 1-5 received open Hartmann reversal. Cases 6-8 received laparoscopic Hartmann reversal. M: male; F: female; BMI: body mass index; ASA: American Society of Anesthesiologists; HR: Hartmann reversal; O: means the patient has the disease; X: means the patient did not have the disease.

	Case 1	Case 2	Case 3	Case 4	Case 5	Case 6	Case 7	Case 8
Age (years)	62	75	42	88	84	65	54	71
Gender	M	M	M	F	F	F	F	M
BMI (kg/m^2^)	28.62	24.06	26.53	27.89	32.49	22.8	23.14	26.03
ASA classification	III	III	III	III	III	III	III	III
Comorbidities								
Cardiovascular disease	O	O	X	O	O	O	O	O
Diabetes mellitus	X	O	X	X	X	O	X	X
End-stage renal disease	X	X	X	X	X	X	X	X
Autoimmune disease	X	X	X	X	X	X	O	X
Liver cirrhosis	X	X	O	X	O	X	X	X
Human immunodeficiency virus	X	X	X	X	X	X	X	X
Cause of initial operation	Benign	Benign	Benign	Benign	Malignant	Benign	Benign	Benign
Median interval duration before HR (months)	3.3	4.2	8.7	4.9	3.8	3.1	2.8	3.2
Hernia	Midline	Stoma	Midline	Midline & stoma	Midline & stoma	Umbilicus & stoma	Umbilicus & stoma	Umbilicus & stoma
Methods of hernia repair	Open	Open	Open	Open	-	Scope	Scope	Scope

Among the five cases in the OHR group, two cases had both midline and stoma hernias, one had only a stoma site hernia, and two had only midline hernias. Cases 6-8 were all diagnosed with both umbilical and stoma site hernias. Four out of the five patients in the OHR group received hernioplasty, while all patients in the LHR group received laparoscopic hernioplasty. Case 5, the only malignant case in the eight patients, did not receive an operation, despite being diagnosed with midline and stoma hernias. The median interval duration before HR was longer in cases 1-5 compared to cases 6-8.

## Discussion

This study demonstrated that in patients who received HR at our institution, the LHR group had significantly shorter length of hospital stay (OHR vs. LHR: 15.00 and 11.50 days, p = 0.028) and faster bowel movement recovery (OHR vs. LHR: 8.00 and 5.00 days, p = 0.022). Furthermore, we observed no significant differences between groups in early and late postoperative complications. The findings might provide surgeons and patients with salutary information when making decisions on these two surgical approaches.

HO was initially developed for rectal cancer management, but advancements in cancer treatments have reduced its necessity in advanced colon carcinoma cases [3]. Currently, HO is mainly applied for benign emergencies like iatrogenic perforation, diverticulitis rupture, or ischemic colitis [5-7]. With these conditions increasingly managed conservatively, HO has become a rare procedure. Moreover, previous studies showed varying reversal rates from 19% to 65%; our study's reversal rate was 32%, consistent with these findings, attributing to the decline in HO cases [8,9].

The demographics and clinical characteristics of both open and laparoscopic groups were similar in age, gender, BMI, ASA score, comorbidities, and the median interval duration before HR. Unlike other reports, patients with malignancy accounted for about 14-43%, and the proportion of malignancy cases in our study was lower, which constituted 9.1% of all patients who received HR [1,10]. At our institution, some surgeons were prone to resect the malignancy and perform primary anastomosis with protective proximal loop stoma (either loop ileostomy or loop transverse colostomy) simultaneously. This might be the reason why we had a lower proportion of malignancy.

Our study found no significant difference in operation time between the two groups, contrasting with most single-center studies, which reported substantial differences and favored the LHR [1,4,11]. For instance, Guerra et al. conducted a meta-analysis in 2019, disclosing that LHR required less time than OHR [12]. The potential reason for the non-significance of our results was that some surgeons routinely fixed the distal colon stump in HO, which might simplify subsequent OHR procedures.

Numerous published data reported significantly lower blood loss in the LHR group [1,12-15]. However, our study showed no significant difference in blood loss between the two groups (p = 0.26). It is worth mentioning that our research exhibited less average blood loss (LHR: 90 ml; OHR: 152.1 ml), even half of the minimal average compared to other studies. Reviewing other articles, the minimum average of estimated blood loss in the LHR group was 166.6 ml, while in the OHR group was 301.1 ml [4,11]. Additionally, most studies did not mention using energy devices except for one study by De’angelis et al. [4]. In our hospital, energy devices that could markedly decrease blood loss were frequently applied for both LHR and OHR groups, which would result in the most negligible blood loss among all the reports.

Contrary to the previous reports (mean score of the OHR group: 2.75, LHR group: 1.38, p = 0.037), the postoperative pain scores did not show significant differences between the two groups, possibly due to variations in analgesia management and the subjective nature of pain assessment [1]. Furthermore, we calculated the average of the highest pain score on days zero, one, three, and seven, which might not represent the actual intensity of pain when we recorded it. Our study indicated that the LHR group benefitted from shorter hospital stays and faster transitions to solid diets, likely facilitated by smaller incisions and quicker recovery of bowel function, consistent with other studies [1,4,11].

Early postoperative complications were analyzed, and no significant differences between the two groups were observed. However, the incidence of early postoperative complications tended toward the open group (OHR group: 39.13%; LHR group: 20%, p = 0.43). We reported two cases of lower gastrointestinal bleeding postoperatively in the OHR group. One case had dark red secretion when the enema tube was removed from the anus on postoperative day three. The enema tube was initially inserted and passed through the anastomosis at the end of HR. It aimed to lower intraluminal pressure and decrease the possibility of leakage. The dark red secretion subsided after conservative treatment. The other patient was recorded to have chronic ulcerative colitis preoperatively. He suffered from two episodes of lower gastrointestinal bleeding and suspected ulcerative colitis flare-ups, which happened two and nine months after HR. We classified the episodes as one early and one late complication for this patient (shown in Table 4). Lower gastrointestinal bleeding was not described in any of the previous reports.

Early postoperative complications, excluding wound infection, ileus, and gastrointestinal bleeding, were classified into "others." In our study, two cases were classified into this category. One case suffered from ischemic cardiomyopathy about two months after HR. Only two studies disclosed patients with cardiac problems after the operation. Cho et al. reported one case that underwent LHR with angina, and Mazeh et al. reported one case that underwent OHR with arrhythmia postoperatively [1,11]. The other case in our study had the underlying disease of myeloproliferative disorder with thrombocytosis. She experienced anemia about four months later and fever with herpes zoster infection six months after the operation.

The most common late complication was incisional hernia (including stoma site hernia, umbilical hernia, and midline incisional hernia). Only one recent study concluded that no significant difference was observed between the two groups, though the LHR group seemed to have a higher incidence of incisional hernia (20%) [1]. In our research, the incisional hernia was defined as the mention of a medical record or from an imaging study. Although there were no significant differences, our study yielded a similar result: the LHR group (30%) seemed to develop more incisional hernia than the OHR group (21.74%). We listed the eight cases with incisional hernias; their details are presented in Table 5. Although the sample size was quite small to calculate statistical significance, a trend showed that patients encountering incisional hernias were those of older age in both groups. Moreover, the data also suggested that patients who underwent OHR with incisional hernias might have a higher BMI. Accordingly, it is important to educate these patients to avoid weight-bearing and encourage them to use abdominal binders. More invasive prophylactic mesh reinforcement might be taken into consideration when patients have risk factors such as the presence of an abdominal aortic aneurysm, obesity, or colorectal surgery [16]. In contrast to the OHR group, all of the patients with incisional hernias in the LHR group received hernioplasty laparoscopically. This could be due to their satisfaction with previous surgeries, including two times of laparoscopic techniques. The faster recovery and more minor wounds from the prior surgeries also increased the willingness of patients to undergo laparoscopic hernioplasty.

We reported one case of postoperative anastomosis stricture. This patient suffered from abdominal fullness after LHR. The lower gastrointestinal series confirmed the diagnosis. Six months following LHR, a further operation involving resection of the stricture site with re-anastomosis was performed. Anastomosis stricture was only mentioned by Part et al. in the table, but no further details were discussed [17].

Anastomosis leakage and mortality were always significant considerations for all surgeons. No anastomosis leakage or mortality was noticed in our research. Upon reviewing previous articles, the rates of anastomosis leakage varied from 4% to 6% [1,10,17-19]. After primary anastomosis, an air leak test was applied to all patients in our institution. When the air leak test was positive, we conducted enhancing sutures near the anastomosis. This procedure could promote a lower leakage rate compared to other studies. Although our study did not capture any mortality for comparison, the results among nearly all studies indicated that the two groups did not exhibit statistical significance in overall mortality [13,20].

The primary limitation of our study is its retrospective design and the small number of participants. Additional constraints include variability in patient factors such as nutritional status, medication history, and the differing technical expertise and experience of surgeons. Additionally, the choice of surgical approach was influenced not only by surgeon preference but also by patient decisions. Despite these limitations, the impact may be partially mitigated by the homogeneity of the patient samples in both the OHR and LHR groups, which supports a more reliable and balanced comparison.

## Conclusions

In conclusion, LHR could enhance quality of life with proper patient selection and minimally invasive techniques, resulting in shorter hospital stays and faster bowel recovery. This study underscores the importance of tailored surgical approaches based on individual factors and institutional practices. When patients are carefully chosen, LHR would be a safe method with low risks of complications and mortality. Future research should focus on prospective studies to strengthen the comparative outcomes between OHR and LHR.
